# GSK-126 Protects CA1 Neurons from H3K27me3-Mediated Apoptosis in Cerebral Ischemia

**DOI:** 10.1007/s12035-021-02677-3

**Published:** 2022-01-29

**Authors:** Zhongcheng Wang, Yaxin Su, Lei Zhang, Ting Lan, Li Li, Suhua Qi

**Affiliations:** 1 Department of Pathophysiology, School of Basic Medical Sciences, Xuzhou Medical University, Xuzhou, China; 2 Laboratory of Clinical and Experimental Pathology, School of Basic Medical Sciences, Xuzhou Medical University, Xuzhou, China; 3 Department of Clinical Laboratory Diagnostics, School of Medical Technology, Xuzhou Medical University, Xuzhou, China

**Keywords:** H3K27me3, GSK-126, Apoptosis, Neuroprotection, Global ischemia stroke

## Abstract

Epigenetics, including histone modifications, play a significant role in central nervous system diseases, but the underlying mechanism remains to be elucidated. The aim of this study was to evaluate the role of H3K27me3 in regulating transcriptomic and pathogenic mechanisms following global ischemic stroke. Here, we found that in vivo ischemic/reperfusion (I/R) injury induced marked upregulation of H3K27me3 in the hippocampus. The administration of GSK-126 to rat brains decreased the levels of H3K27me3 in the hippocampus and reduced neuronal apoptosis after experimental stroke. Furthermore, ChIP-seq data demonstrated that the primary role of GSK-126 in the ischemic brain is to reduce H3K27me3 enrichment, mediating negative regulation of the execution phase of apoptosis and the MAPK signaling pathway. Further study suggested that the protective role of GSK-126 in ischemic rats was antagonized by U0126, an inhibitor of ERK1/2. Collectively, we demonstrated the potential of H3K27me3 as a novel stroke therapeutic target, and GSK-126 exerted a neuroprotective function in ischemic brain injury, which might be associated with activation of the MAPK/ERK pathway.

**Graphical Abstract:**

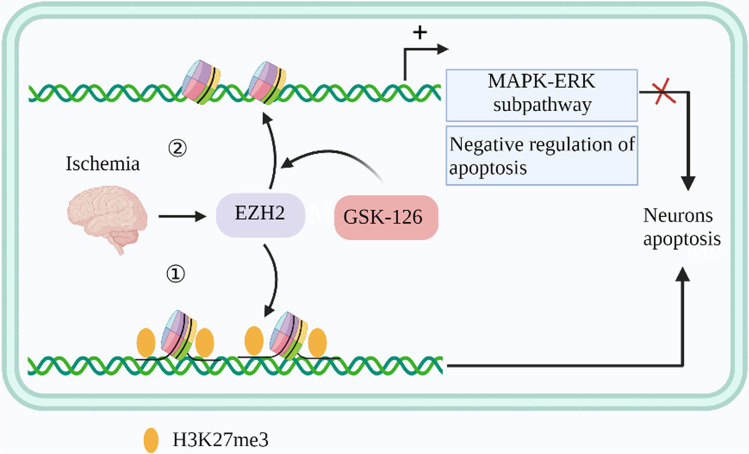

**Supplementary Information:**

The online version contains supplementary material available at 10.1007/s12035-021-02677-3.

## Introduction

Stroke is one of the leading causes of death and disability in the world, approximately 87% of which is ischemic stroke [1]. Both ischemic and hemorrhagic stroke lead to neuronal death. In response to global cerebral ischemia, hippocampal pyramidal neurons in the cornu ammonis 1 (CA1) region are particularly vulnerable, and apoptosis in the hippocampal CA1 region is the major cause of cell death [2]. Recognized mechanisms of neuronal death in stroke include excitotoxicity, calcium overload, and oxidative stress, but whether histone methylation, especially H3K27me3, participates in these processes is still uncertain.

Emerging roles suggest that abnormal H3K27me3 is found in many central nervous system diseases [3]. H3K27me3 is an epigenetic gene repressor that plays a critical role in many neuronal processes, including neurodegeneration, synaptic function, epilepsy, and animal behavior [4–6]. Stroke is a disease of aging, and H3K27me3 levels increase with age [7, 8]. Investigation into the relationship between H3K27me3 and stroke has increased. H3K27me3 is mediated by enhancer of zeste homolog-2 (EZH2), a histone methyltransferase of the catalytic component of polycomb repressor complex-2 (PCR2). Currently, few studies on EZH2/H3K27me3 in the prognosis or progression of stroke have involved neurons. As previously reported, inhibition of EZH2 activity promotes the differentiation of human plasma stem cells into neurons by increasing peroxisome proliferator-activated receptor gamma (PPARɣ) expression, and transplantation of human plasma stem cells with EZH2 knockdown into the ischemic brain promotes functional recovery from ischemic brain injury [9]. Moreover, EZH2 may regulate the activation of microglia by activating phosphorylated signal transducer and activator of transcription 3 (STAT3), aggravating the inflammatory response after ischemic stroke. The EZH2 inhibitor 3-deazaneplanocin A (DZnep) exerts a neuroprotective effect after ischemic stroke [10]. Another EZH2 inhibitor, GSK-126, was also found to be safe for use in the central nervous system [11]. Therefore, in the present study, we investigated whether H3K27me3 was involved in the risk of stroke and whether GSK-126 may be used for the prevention and treatment of stroke.

In brief, the relationship between H3K27me3 and neuronal death in the ischemic brain was characterized in the present study. In addition, this study also revealed that H3K27me3 may participate in the regulation of apoptosis through the MAPK pathway in the ischemic brain. These findings are not only beneficial for us to understand the role of H3K27me3 in the pathogenesis of stroke but also suggest the potential value of GSK-126 in the prevention and treatment of stroke.

## Materials and Methods

### Animals and Ethics

Sprague–Dawley rats (male) weighing 230–250 g were obtained from the laboratory animal center of Xuzhou Medical University. The animals were housed in groups of 4–5 rats/cage with free access to food and water under a 12 light/12 dark cycle. In the present study, rats were randomly assigned to the experimental groups. All animal procedures were approved by the committee on the ethics of animal experiments of Xuzhou Medical University (D2016017), and animals were cared for in accordance with the Guide for the Care and Use of Laboratory Animals.

### Preparation of the Transient Global Cerebral Ischemia Model

We utilized a model of global cerebral ischemia and reperfusion induced by four vessel occlusions as previously described [12, 13]. In brief, rats were anesthetized with isoflurane, both vertebral arteries were permanently electrocauterized, and the common carotid arteries were isolated. After 24 h, the common carotid arteries were re-exposed and clipped using artery clips for 15 min followed by reperfusion. During the entire procedure, a heating pad and heating lamps were used to maintain the rectal temperature at 37 °C. Rats that lost their righting reflex within 60 s, those that lost consciousness, and those that exhibited white eyeballs during ischemia were selected for subsequent experiments. Sham-operated control rats were subjected to the same procedure except for occlusion of the carotid artery. After 24 h of ischemia and reperfusion, modified neurological severity score (mNSS) was adopted to evaluate the neurological function of rats in each group [14]. In this study, the animal survival rate was approximately 65–75%, and the model success rate was 75%.

### Drugs and Administration

For the surgery, rats were anesthetized using isoflurane and placed into a stereotactic device with lambda and bregma at a horizontal level. A midline incision of the skin was made to unbar the skull, and a small hole was drilled into the skull using the bregma as a reference (stereotaxic zero) at the following stereotactic coordinates: mediolateral (ML): 1.0 mm away from midline on the left side; anteroposterior (AP): 1.5 mm posterior to the bregma, which corresponded to the lateral ventricle region, based on the anatomical atlas of the rat brain. The catheter was inserted into the small hole and secured using dental cement. To eliminate inflammation and repair the blood-brain barrier, the rats were placed back into their cages and allowed to rest for 3 days. For the intracerebroventricular injection (i.c.v.), rats were anesthetized using isoflurane, and drugs or normal saline were injected starting on the fourth day.

GSK-126 and U0126 were obtained from Vicmed Biotech (Xuzhou, China). The powder formulations were stored at −20 °C and dissolved in 20% Captisol adjusted to pH 4–4.5 using 1 N acetic acid before use in in vivo studies at a final dissolved concentration of 5 mg/ml. GSK-126 was injected into the lateral ventricle for 7 consecutive days (20 μl/day, i.c.v.) before global cerebral ischemia modeling, and U0126 was administered 30 min prior to ischemia (5 μl, 0.2 μg/μl, i.c.v.). Rats in the sham group were administered equal volumes of normal saline. All drugs were injected into the lateral ventricle by using a mini pump (R462, RWD Biotech, Shenzhen, China).

### Western Blot

Rat hippocampal tissues were collected 24 h after reperfusion. Protein samples (40–50 μg in each lane) were loaded onto SDS PAGE gels for electrophoresis. Proteins were transferred to polyvinylidene difluoride membranes and then blocked in 5% dried milk at room temperature. Primary antibodies were added and incubated at 4 °C overnight. H3K27me3 (mAbcam6002) and histone H3 (mAbcam10799) were purchased from Abcam (Shanghai, China), and β-actin (ab179467) and caspase-3 (19677-1-AP) were purchased from Proteintech (Wuhan, China). Then, the membrane was incubated with the secondary antibody (dilution 1:5000) at 37 °C for 2 h. The protein bands were detected using a chemiluminescent substrate kit (Merck Millipore, USA) according to the manufacturer’s recommendations. Band intensities were quantified using Quantity One software.

### Nissl Staining

After 15 min of ischemia and 5 days of reperfusion, the rats were given isoflurane anesthesia and euthanized to remove the intact brain tissue, which was placed in 4% paraformaldehyde and fixed for 12 h. Coronal sections (10-μm thick) were cut and stored at −80 °C. Every hippocampus section was selected for Nissl staining using 0.3% cresyl violet to evaluate infarct volumes.

### TUNEL Staining

After 15 min of ischemia and 24 h of reperfusion, the brain tissues were removed as it was mentioned above. In brief, sections were dewaxed by conventional methods, and then were incubated with 1% Triton-100 for 15 min at room temperature, and subsequently incubated with 3% H_2_O_2_ for 15 min at room temperature. Finally, TUNEL reaction mixture was then added to each sample and then incubated at 37 °C for 60 min.

### Chromatin Immunoprecipitation Sequencing (ChIP-seq) and Data Mining

Hippocampal tissues were collected after 24 h of reperfusion. ChIP analysis was performed using a chromatin immunoprecipitation kit (Millipore, USA) according to the manufacturer’s instructions. Immunoprecipitated and input DNA was purified and transferred to CloudSeq Biotech (Shanghai, China) to perform high-throughput sequencing. Raw data were generated after sequencing, and image analysis, base calling, and quality filtering were performed on an Illumina NovaSeq 6000 sequencer. Peak calling was performed using MACS software. Differentially enriched regions (*p* < 0.05) were identified using diffReps software. The enriched peaks were then annotated using the latest UCSC RefSeq database to link the peak information to the gene annotation.

Gene ontology (GO) and pathway analyses were performed on the peak-associated genes or differentially enriched peak-associated genes. GO analysis was performed to elucidate the biological implications of unique genes in the significance experiment. The “elim Fisher” algorithm was used for the GO enrichment test. GO categories with a *p* < 0.01 are reported. The gene ontology is structured as a directed acyclic graph, and each term has defined relationships to one or more other terms. Pathway analysis was implemented to determine the significant pathways of the peak-associated genes according to the KEGG database. Fisher’s exact test was used to identify significant enrichment for pathways; pathway categories with *p* < 0.01 are reported.

### Statistics

All data were analyzed by an investigator blinded to the group allocation. GraphPad Prism 5 was used for all data analysis. Throughout the text, summary data are presented as the means ± SEM. One-way ANOVA and *T*-tests were used to assess the statistical significance of the differences between groups.

## Results

### GSK-126 Decreases H3K27me3 Levels in the Rat Hippocampus

**Fig. 1:**
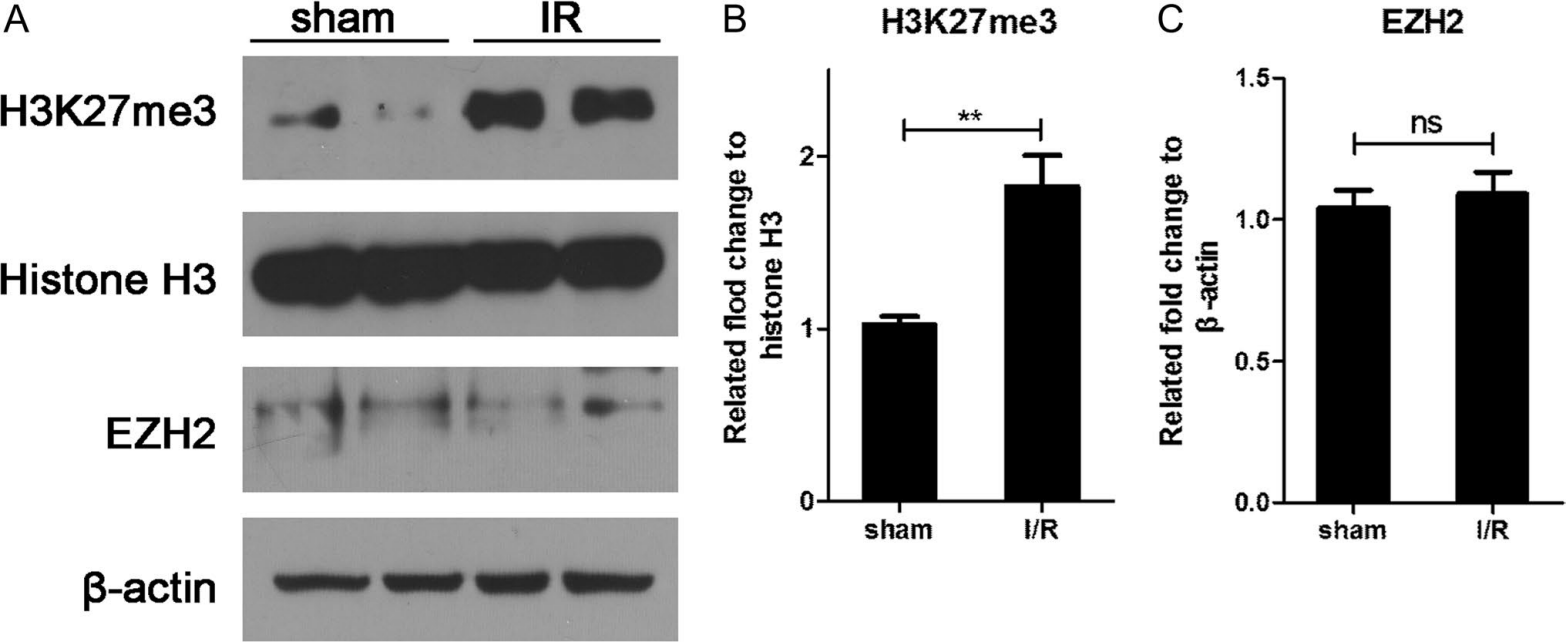
H3K27me3 levels are elevated in the ischemic rat brain. **A** Representative levels of H3K27me3 and EZH2 in I/R rats. Histone H3 and β-actin were used as loading controls. **B** The relative intensity of H3K27me3 illustrated in panel **A**. **C** The relative intensity of EZH2 illustrated in panel **A**. **p < 0.01

**Fig. 2:**
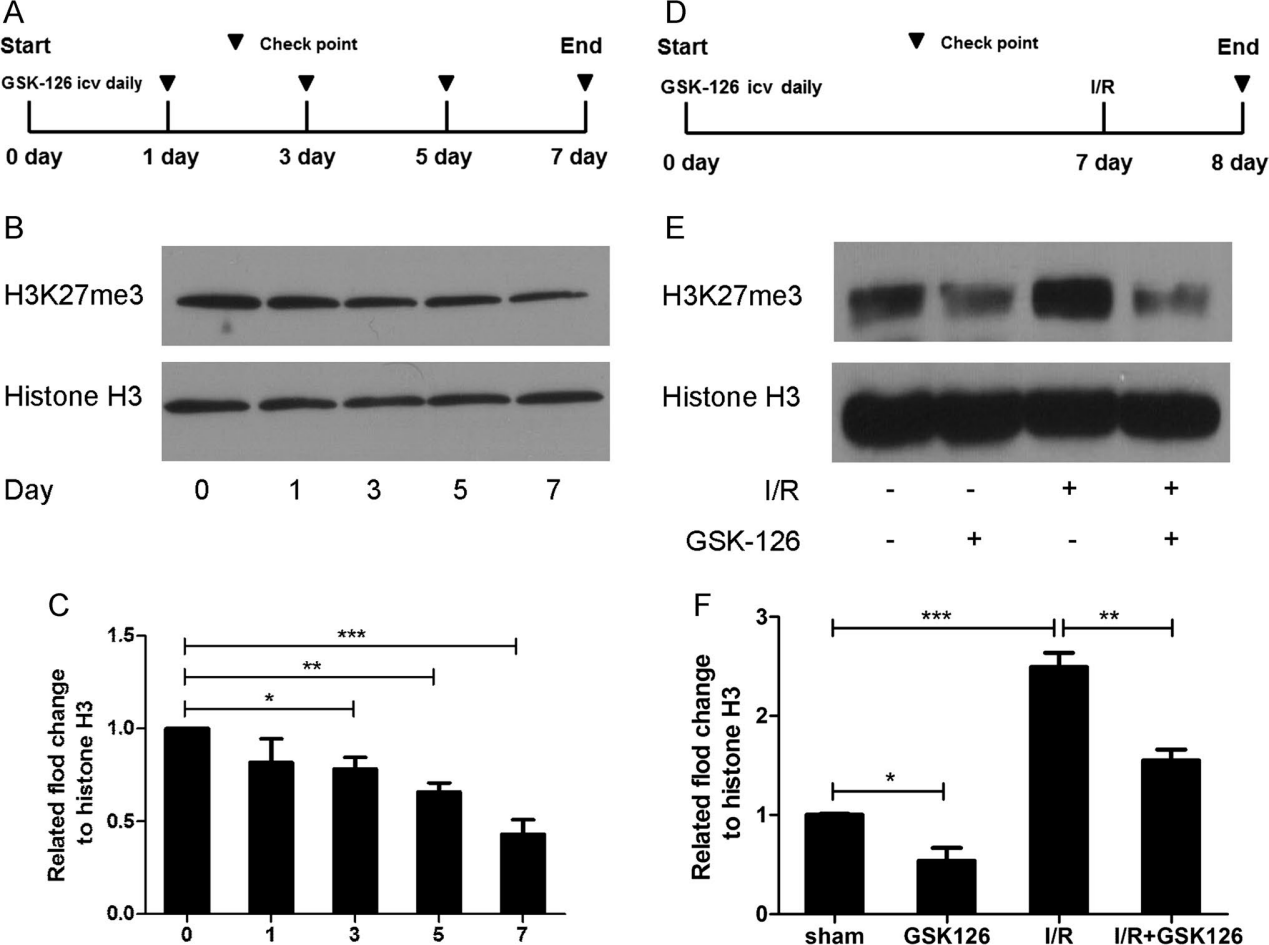
GSK-126 reduces levels of H3K27me3 in the rat hippocampus in response to ischemia. **A** Schematic diagram of the time course experiment. **B** Protein extracts from the rat hippocampus were probed by western blotting with H3K27me3 antibodies. Histone H3 was used as a loading control. Protein levels of H3K27me3 decreased after 7 consecutive days of injection, and GSK-126 was injected for 7 consecutive days in subsequent experiments. **C** Relative intensity of H3K27me3 illustrated in panel **B**. **D** Schematic diagram of GSK-126 administration and global brain ischemia modeling. Global cerebral ischemia model surgery was performed after rats were administered GSK-126 for 7 consecutive days. **E** Compared to I/R rats, I/R rats preadministered GSK-126 exhibited reduced H3K27me3 levels in the hippocampus. Protein extracts from the rat hippocampus were probed on western blots with H3K27me3 antibodies. Histone H3 was used as a loading control. **F** Relative intensity of H3K27me3 illustrated in panel **E**. *p < 0.05. **p < 0.01. ***p < 0.001. *N* = 3

We speculated that during the ischemia reperfusion process, levels of H3K27me3 increased in the brain. To test this hypothesis, we initially examined whether ischemia increases H3K27me3 in the hippocampus. A remarkable increase in the levels of H3K27me3 was observed in the I/R groups, and this increase in H3K27me3 was not due to abnormal EZH2 expression (Fig. 1A–C). To determine whether GSK-126 decreased the levels of H3K27me3 in the hippocampus, we next performed a time effect test. As shown in Fig. 2A–C, levels of H3K27me3 in the hippocampus were successfully reduced by i.c.v. injection of GSK-126 for 7 consecutive days. Therefore, the global cerebral ischemia model surgery was performed 7 days after injection of GSK-126 (Fig. 2D). Moreover, compared to the I/R group, rats preadministered GSK-126 exhibited reduced H3K27me3 levels in response to ischemia induction (Fig. 2E–F).

### GSK-126 Protects CA1 Neuron Survival in Response to Ischemia

**Fig. 3:**
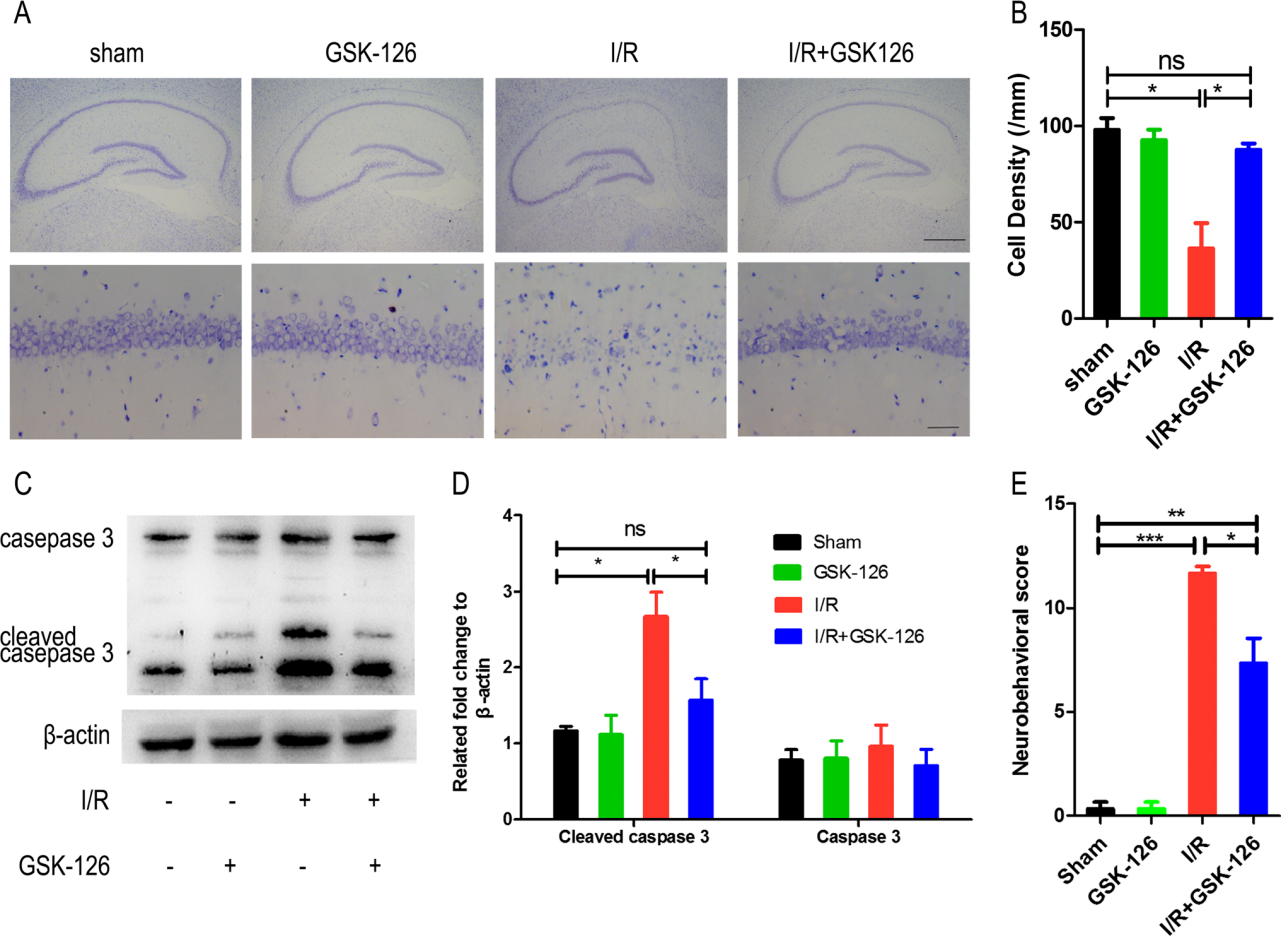
GSK-126 decreases CA1 neuronal apoptosis after ischemia. **A** Nissl staining was performed on rat hippocampal sections. The boxed areas (CA1 areas) in the upper row are shown at higher magnification in the second row. The scale bar is 500 mm in the top line and 50 μm in the bottom line. **B** Quantitative analysis of the above data, which were obtained from three rats in each group, and the results of a typical experiment are presented. **C** Representative levels of caspase-3 and cleaved caspase-3 in each group. Protein extracts from the rat hippocampus were probed on western blots using caspase-3/cleaved caspase-3 antibodies. β-actin was used as a loading control. **D** Relative intensity of caspase-3 and cleaved caspase-3 illustrated in panel **C**. **E** The behavioral evaluation by the mNSS test. *p < 0.05, **p < 0.01, ***p < 0.001, ns indicates no significant differences. *N* = 6

To determine the effect of GSK-126 on brain ischemia and reperfusion, Nissl staining was performed to detect changes in the number of hippocampal neurons. As shown in Fig. 3A–B, pyramidal neurons in the CA1 region were particularly vulnerable to ischemia, but GSK-126 protected CA1 neurons from ischemia. GSK-126 itself did not affect the arrangement, shape, or conformation of pyramidal cells in the CA1 region. As apoptosis in the hippocampal CA1 region is the major cause of cell death, we next examined whether GSK-126 reduced CA1 neurons apoptosis. A significant increase in the level of cleaved caspase-3, but not caspase-3, was observed in ischemic rats, and which could be reversed by GSK-126 (Fig. 3C–D). Combined with the TUNEL staining (Fig. S1), we could conclude that protective role of GSK-126 on CA1 neurons after I/R was due to its inhibitory effect on apoptosis. In addition, according to the mNSS score, GSK-126 can improve the behavioral performance in ischemic rats (Fig. 3E). In brief, this result demonstrated that GSK-126 has potential as a treatment for stroke.

### GSK-126 May Inhibit Apoptosis via the MAPK Pathway in the Ischemic Brain

**Fig. 4:**
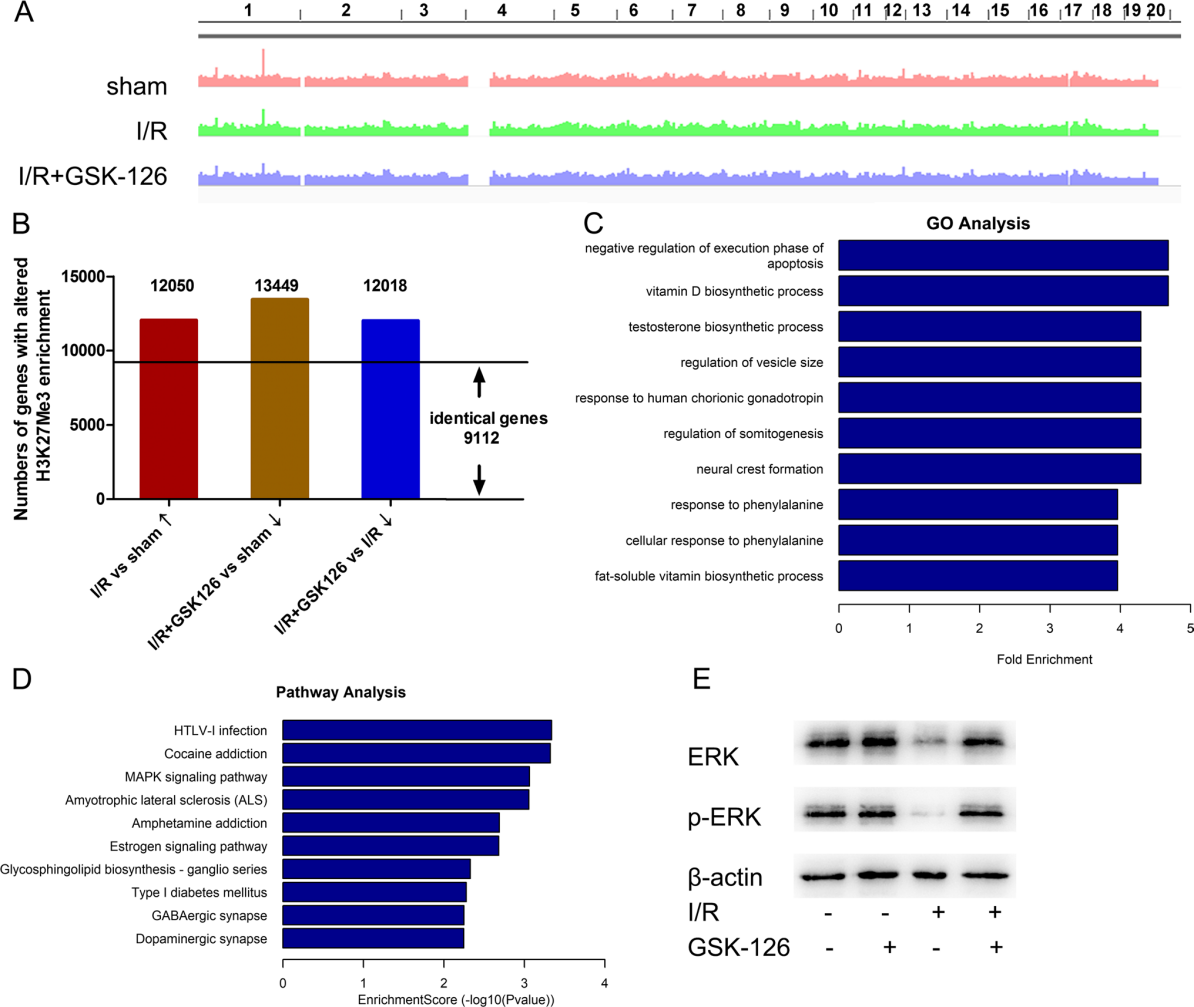
ChIP-seq analysis. **A** An illustration of H3K27me3 enrichment in the entire genome. **B** Numbers of genes with differential enrichment of H3K27me3 in each group. The arrow after the group name indicates upregulated or downregulated enrichment levels of H3K27me3 in the specified group. **C** Significant GO category for genes with downregulated enrichment levels of H3K27me3 between I/R rats pretreated with GSK-126 and I/R rats. **D** Top 10 significant pathways for genes with downregulated enrichment levels of H3K27me3 between the I/R rats pretreated with GSK-126 and I/R rats. **E** Representative levels of ERK and p-ERK in each group. Protein extracts from the rat hippocampus were probed on western blots using ERK and p-ERK antibodies, respectively. β-actin was used as a loading control

To further determine the mechanisms of GSK-126 in protecting neuronal survival in response to ischemia, ChIP-seq was performed to determine the variation in H3K27me3 enrichment in the whole genome (Fig. 4A). As the role of GSK-126 was to inhibit levels of H3K27me3, we were primarily concerned about the differences between ischemic rats preadministered GSK-126 and control ischemic rats. Due to the extensive inhibitory effects of H3K27me3 on gene expression, increased H3K27me3 was found to be enriched in 12050 gene sequences in ischemic rats compared to sham rats, while decreased H3K27me3 levels were found to be enriched in 13449 gene sequences in ischemic rats pretreated with GSK-126 compared to sham rats. Furthermore, decreased H3K27me3 enrichment was found on 12108 genes in ischemic rats pretreated with GSK-126 compared to ischemic rats. However, these three groups shared 9112 genes in common, suggesting that the increased enrichment of H3K27me3 on most genes caused by ischemia could be reversed by GSK-126 (Fig. 4B). We speculate that genes upregulated in ischemic rats preadministered GSK-126 are responsible for the neuroprotective effects of GSK-126. Therefore, H3K27me3 should be less enriched on those gene sequences. The GO category results confirmed this hypothesis. In contrast to ischemic rats, the most significantly different GO terms for genes with reduced H3K27me3 enrichment in I/R rats pretreated with GSK-126 compared to I/R rats were associated with negative regulation of apoptosis (Fig. 4C, Table S1). In addition, pathway analysis of the above genes revealed a remarkable decrease in H3K27me3 enrichment in genes involved in the MAPK pathway (Fig. 4D, Fig. S2A, Table S2). MAPKs mediate intracellular signaling pathways associated with cell death and survival in stroke, which has been demonstrated by numerous studies [15, 16]. Whether GSK-126 protects neurons from apoptosis by activating the MAPK pathway is a hypothesis worth exploring.

### U0126 Antagonizes the Antiapoptotic Effect of GSK-126 in the Ischemia/Reperfusion Brain

**Fig. 5:**
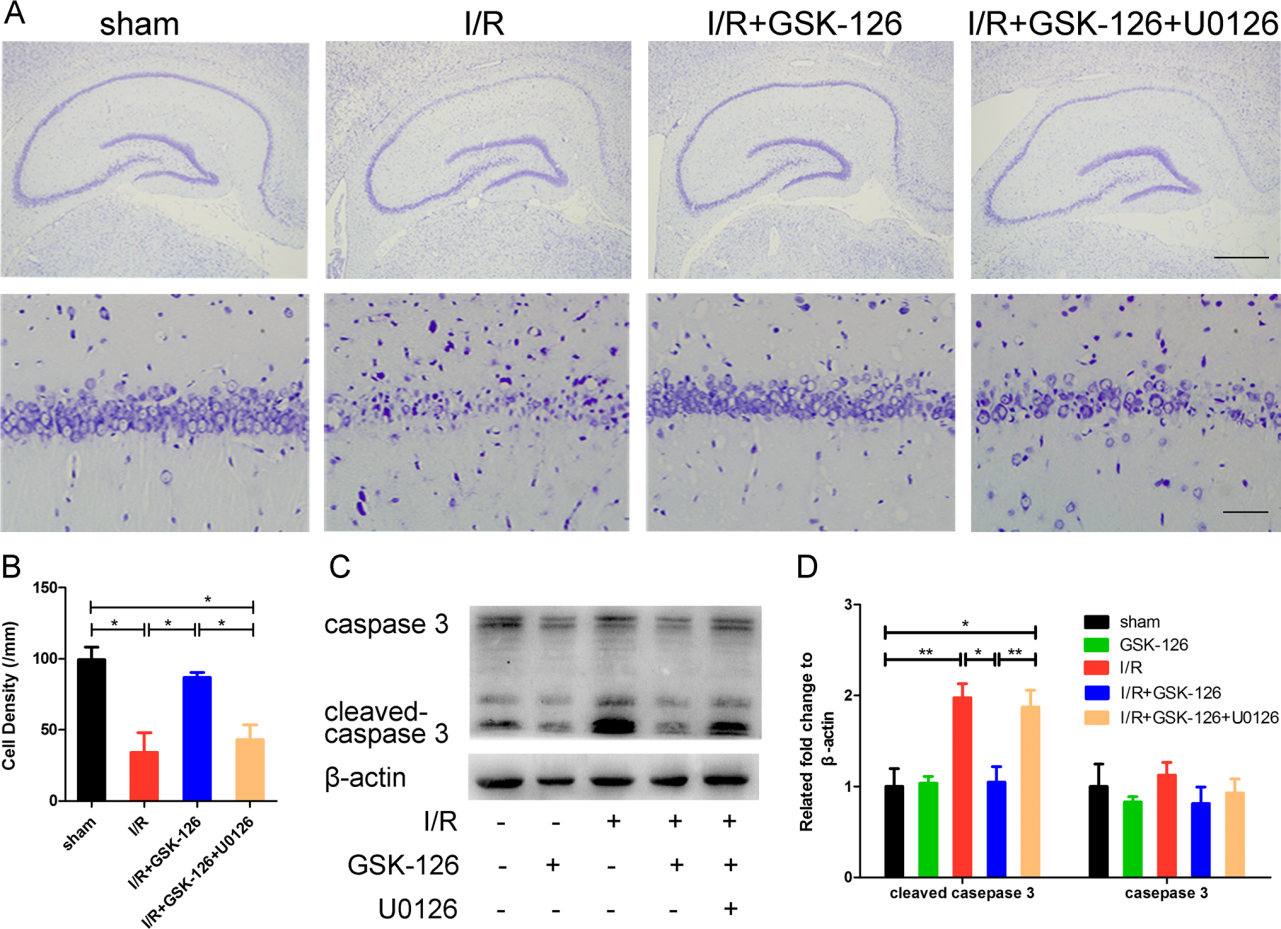
U0126 antagonizes the protective effect of GSK-126 in the I/R brain. **A** Nissl staining was performed on rat hippocampal sections. The upper line shows neurons in the hippocampus of each group. The boxed areas (CA1 areas) in the upper line are higher magnification of the bottom line. Scale bar, 50 μm. **B** Quantitative analysis of the above data, which were obtained from three rats in each group, and the results of a typical experiment are presented. **C** Representative levels of caspase-3/cleaved caspase-3 in each group. Protein extracts from the rat hippocampus were probed on western blots using caspase-3/cleaved caspase-3 antibodies. β-actin was used as a loading control. **D** Relative intensity of caspase-3 and cleaved caspase-3 illustrated in panel **C**. *p < 0.05, **p < 0.01. *N* = 3

Among the MAPK pathway genes mentioned above, several key genes of the MAPK/ERK1 signaling pathway were involved (Table S2). The extracellular signal-regulated kinase 1/2 (ERK1/2) signaling cascade plays a vital role in promoting neuronal survival during focal and global brain ischemic injury, and U0126 is a specific inhibitor of ERK1/2 [17–19]. To investigate whether GSK-126 protects neurons from apoptosis by activating the ERK1 pathway, we first examined whether GSK-126 treatment modulating ERK activity. As it was shown in Fig. 4E, significant increase in the levels of p-ERK and ERK was observed in ischemic rats that were treated with GSK-126. The interesting thing is that the increasing level of p-ERK was highly associated with the expression level of ERK (Fig. 4E, Fig. S3). To provide the evidence to verify that ERK pathway was critical to GSK-126 to protect CA1 neurons after I/R, U0126 was administered to rats pretreated with GSK-126 before ischemia. The protective role of GSK-126 in ischemic rats was antagonized by U0126 (Fig. 5, Fig. S1, and Fig. S4). Consistent with the Nissl’s staining result, a significant increase in cleaved caspase-3 was observed in ischemic rats, which was reversed by pretreatment GSK-126. However, the reduction in cleaved caspase-3 caused by GSK-126 after ischemia was antagonized by the administration of U0126. Moreover, changes in the levels of cleaved caspase-3 were independent of the expression of caspase-3 (Fig. 5C–D). In brief, U0126 antagonizes the antiapoptotic effect of GSK-126 in animal models of cerebral ischemia.

## Discussion

**Fig. 6:**
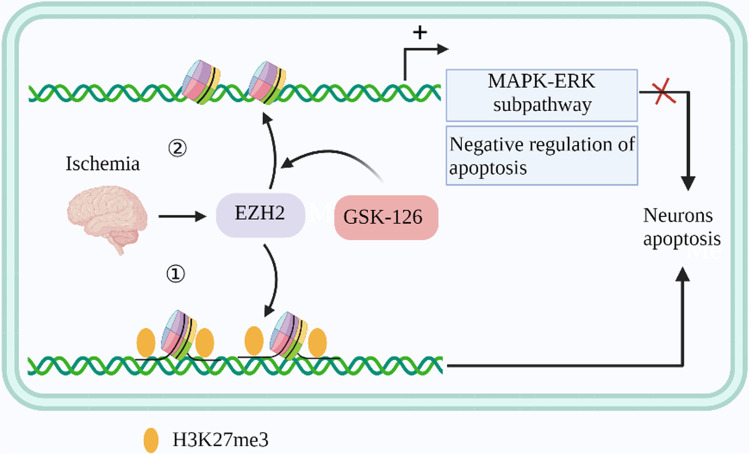
A schematic illustration of the possible mechanism by which GSK-126 protects neurons from apoptosis. **①** After ischemic stroke, the levels of H3K27me3 are increased by EZH2, leading to neuronal apoptosis. **②** GSK-126 reduces the expression levels of H3K27me3 by interfering with the activity of EZH2 and reduces the enrichment of H3K27me3 on gene sequences, including genes related to negative regulation of apoptosis and the MAPK pathway. These genes are helpful for inhibiting neuronal apoptosis. Moreover, U0126 targets EK1/2 to antagonize the effects of GSK-126 in preserving neurons in the ischemic brain. The orange plot indicates H3K27me3

Ischemic stroke is initiated by the interruption of cerebral blood flow and is followed by reperfusion, which further induces neuronal death, leading to brain tissue injury. Apoptosis is the primary mechanism of cell death and is regulated by several signaling pathways [20]. Investigation of novel treatment strategies to target cell death is still needed. Related studies have found that histone methylase inhibitors and histone demethylase inhibitors have effects in the treatment of ischemic stroke [21]. However, they have rarely been reported to be related to the apoptotic pathway of ischemic stroke. In this study, we demonstrated that the EZH2 inhibitor GSK-126 alleviated neuronal apoptosis induced by ischemic stroke. Moreover, MAPK/ERK pathway activation was required for this process (Fig. 6).

Methylation levels of H3K27 are mediated by the histone methylase EZH2 and the histone demethylase JMJD3. H3K27me3 is involved in a variety of biological processes by inhibiting gene expression. However, the role of H3K27me3 in neuronal death has been less well investigated. A previous report revealed that JMJD3 rescues apoptosis in hippocampal neurons by upregulating the expression of BDNF, and JMJD3 plays an important role in the expression of Bax and caspase-3 during oxygen–glucose deprivation (OGD) injury [22, 23]. In addition, we previously found that elevation of H3K27me3 downregulated the expression of Na^+^/Ca^2+^ exchanger 3 (NCX3) in hippocampal neurons, while GSK-126 restored expression levels of NCX3 [11]. NCX3 is a gene that reduces neuronal death after ischemic brain damage [24]. Combined with our observation in I/R rats, these results indicate that the increasing H3K27me3 levels in the brains of I/R rats were independent of the expression of EZH2 (Fig. 1). It would be interesting to examine whether an EZH2 inhibitor such as GSK-126 protects neurons from ischemic brain injury-induced neuron death.

GSK-126 was originally developed as a more effective inhibitor of EZH2 [25]. It exhibits higher selective inhibition of EZH2 than other EZH2 inhibitors, such as DZNep and EPZ005687 [26]. Moreover, GSK-126 has entered phase I clinical trials [27]. However, low oral bioavailability and blood-brain barrier permeability issues with GSK-126 were observed in animal models, which may limit its use in the central nervous system [28]. In contrast, reports have suggested that large doses of intraperitoneally administered GSK-126 reduce levels of H3K27me3 in the brain [11]. Therefore, to investigate the role of H3K27me3 in the ischemic brain, GSK-126 was administered to rats by intracerebroventricular injection in the present study. A time course experiment was performed and revealed that levels of H3K27me3 in the hippocampus were reduced in response to 7 consecutive days of intracerebroventricular injection of GSK-126 (Fig. 2A–C). Rats preadministered GSK-126 exhibited attenuated H3K27me3 levels (Fig. 2D–F) and more surviving neurons in response to I/R (Fig. 3).

The mechanism by which GSK-126 affects neuronal death is still unknown. According to the ChIP-seq analysis, the essential genes bound and differentially enriched by H3K27me3 span a multitude of various genes (Fig. 4A). Because GSK-126 inhibited levels of H3K27me3, we primarily analyzed the difference in H3K27me3 enrichment between I/R rats preadministered GSK-126 and I/R rats, along with control animals. Interestingly, we found that the three groups shared 9112 identical genes (Fig. 4B). These identical genes indicate that most genes affected by increasing levels of H3K27me3 after ischemia were reversed by GSK-126. Therefore, GO analyses were performed to detect the potential relationship between these genes and the pathology of ischemic stroke. The results revealed that negative regulation of the execution phase of apoptosis was the most significantly altered GO, and compared to ischemic rats, ischemic rats preadministered GSK-126 negatively regulated antiapoptotic genes (DFFA, NMAT1, PAM16, BCL2L1, FZD3, and CXCR3) (Fig. 4C, Table S1). Among these genes, NMAT1 and BCL2L1 are responsible for reducing cell death and apoptosis after ischemic stroke [29, 30]. CXCR3, the receptor of CXCL10, is known to reduce brain infarction and attenuate BBB disruption in stroke [31]. In addition, the known causes of neuronal apoptosis after stroke include reactive oxygen species (ROS), calcium overload, excitatory toxicity, and inflammatory *reactions*. ROS are one of the early and most important components of cerebral brain ischemia, and excessive production of ROS leads to oxidative injury, including DNA damage, protein oxidation, and lipid peroxidation, ultimately leading to apoptosis [32]. After ischemic stroke, ATP deficiency leads to Ca^2+^ overload, which can induce neuronal apoptosis [33, 34]. Moreover, cerebral ischemia results in release of large amounts of glutamate, which stimulates NMDARs and induces calcium influx through these ionotropic receptors. The calcium-dependent activation of death-signaling proteins that are immediately downstream of the receptors triggers excessive signaling cascades that work cooperatively to induce neuronal death [35]. Therefore, excitotoxicity is considered one of the most important mechanisms of neuronal apoptosis after ischemia [36]. In the present study, the GO results were consistent with the above research. Altered G protein-coupled glutamate receptor signaling pathways were also identified between sham rats and I/R rats pretreated with GSK-126 (Fig. S2B, Table S3).

The KEGG pathway analysis suggested that the MAPK signaling pathway was one of the most significantly altered pathways. The MAPK pathway consists of three subpathways: ERK-MAPK, JNK-MAPK, and p38-MAPK. ERK1/2, JNK, and p38 can induce both cell survival and cell death in response to ischemic stroke [20]. Among them, ERK1/2 was reported to play an antiapoptotic role in the process of ischemic brain injury, while JNK and p38 promote apoptosis after ischemia [37, 38]. Here, we found that 40 genes in the MAPK pathway were affected by H3K27me3 enrichment (Fig. 4D, Table S2). In addition, several key genes (BDNF, MEKK2, MYC, etc.) in the ERK1/2 pathway were included in these 40 genes. ERK can be activated by BDNF, MEK is an upstream protein of ERK, and MYC is activated by ERK [20, 39, 40]. This evidence suggests that GSK-126 may activate the entire ERK1/2 signaling pathway in the brains of I/R rats.

To test the above hypothesis, rats were administered the MEK inhibitor U0126 after 7 days of GSK-126 injection to block the ERK1/2 pathway before ischemia modeling. As expected, the role of GSK-126 in protecting CA1 neurons from ischemia-induced apoptosis was antagonized by administration of U0126 (Fig. 5, Fig. S1). Levels of cleaved caspase-3 further confirmed this conclusion. Our results indicate that activation of the ERK1/2 pathway may contribute to the reduction of neuronal apoptosis after ischemia. Nevertheless, whether ERK activation promotes or inhibits neuronal apoptosis is still uncertain. Some reports indicate that inhibition of the ERK signaling pathway by U0126 inhibits apoptosis after ischemia [41, 42]. These conflicting results may be due to the complexity of the MAPK signaling pathway and the inconsistency among experimental conditions. In our research, it would be helpful to further study the impact of H3K27me3 on the JNK-MAPK and p38-MAPK pathways.

In addition, the GABAergic synapse pathway was among the top 10 significantly altered pathways in I/R rats preadministered GSK-126 compared to I/R rats (Fig. 4D), which is in line with the findings that GABA has a protective effect in ischemic stroke [43, 44]. During an ischemic episode, the extracellular cerebral GABA concentration increases. Related studies have revealed that GABAergic signaling is enhanced through pharmacological intervention just hours after stroke; neuroprotection can be achieved by reducing the excitotoxic index and lowering the release of glutamate [45, 46]. These reports further validate the accuracy of our ChIP-seq data. Moreover, they also suggest that studying the epigenetic regulation of the GABAergic signaling pathway may contribute to in-depth understanding of these excitotoxicity effects.

In conclusion, we demonstrated that the EZH2 inhibitor GSK-126 alleviates neuronal apoptosis in ischemic stroke by inhibiting H3K27me3. Furthermore, activation of the ERK-MAPK pathway is required for the neuroprotective function of GSK-126 in the ischemic brain.

## Supplementary Information

Supplemental Figure 1. **Tunel assay.** A. Tunel assay was performed on rat hippocampal section. The left panels show the whole hippocampal section of each group, the scale bar is 200μm.The right panels show the CA1 section of each group, the scale bar is 50 μm. Apoptotic cells were labeled with fuorescein isothiocyanate (green), and all nuclei were stained with DAPI (blue). The arrow points to the apoptotic cells. A. Representive section of sham group. B. Representive section of GSK-126 group. C. Representive section of I/R group. D. Representive section of I/R+ GSK-126 group. E. Representive section of I/R+ GSK-126+U0126 group. N=4. (PNG 3650 kb)

High Resolution Image (TIF 110039 kb)

Supplemental Figure 2. **Pathway analysis and GO analysis.** A. The gene ratio dot plot of the top 10 significant pathways. B. The top 10 significant GO terms for genes with downregulated enrichment levels of H3K27me3 between I/R rats pretreated with GSK-126 and sham rats. (PNG 226 kb)

High Resolution Image (TIF 2932 kb)

Supplemental Figure 3. **ERK expreesion level in each group.** Relative intensity of ERK and pERK illustrated in Figure 4E. *p< 0.05, **p < 0.01. N=3. (PNG 18 kb)

High Resolution Image (TIF 67 kb)

Supplemental Figure 4. **Behavioral evaluation by the mNSS test.** ns indicates no significant differences. *p< 0.05, **p < 0.01. N=3. (PNG 24 kb)

High Resolution Image (TIF 131 kb)

ESM 1 (XLS 672 kb)

ESM 2 (XLS 39 kb)

ESM 3 (XLS 1064 kb)

## Data Availability

The data that support the findings of this study are available from the corresponding author upon reasonable request.
