# Supplementary figures and images for: GSK-126 Protects CA1 Neurons from H3K27me3-Mediated Apoptosis in Cerebral Ischemia
Source: Mol Neurobiol. 2022 Jan 29;59(4):2552–62. doi: 10.1007/s12035-021-02677-3 (PMC9016005; doi:10.1007/s12035-021-02677-3)

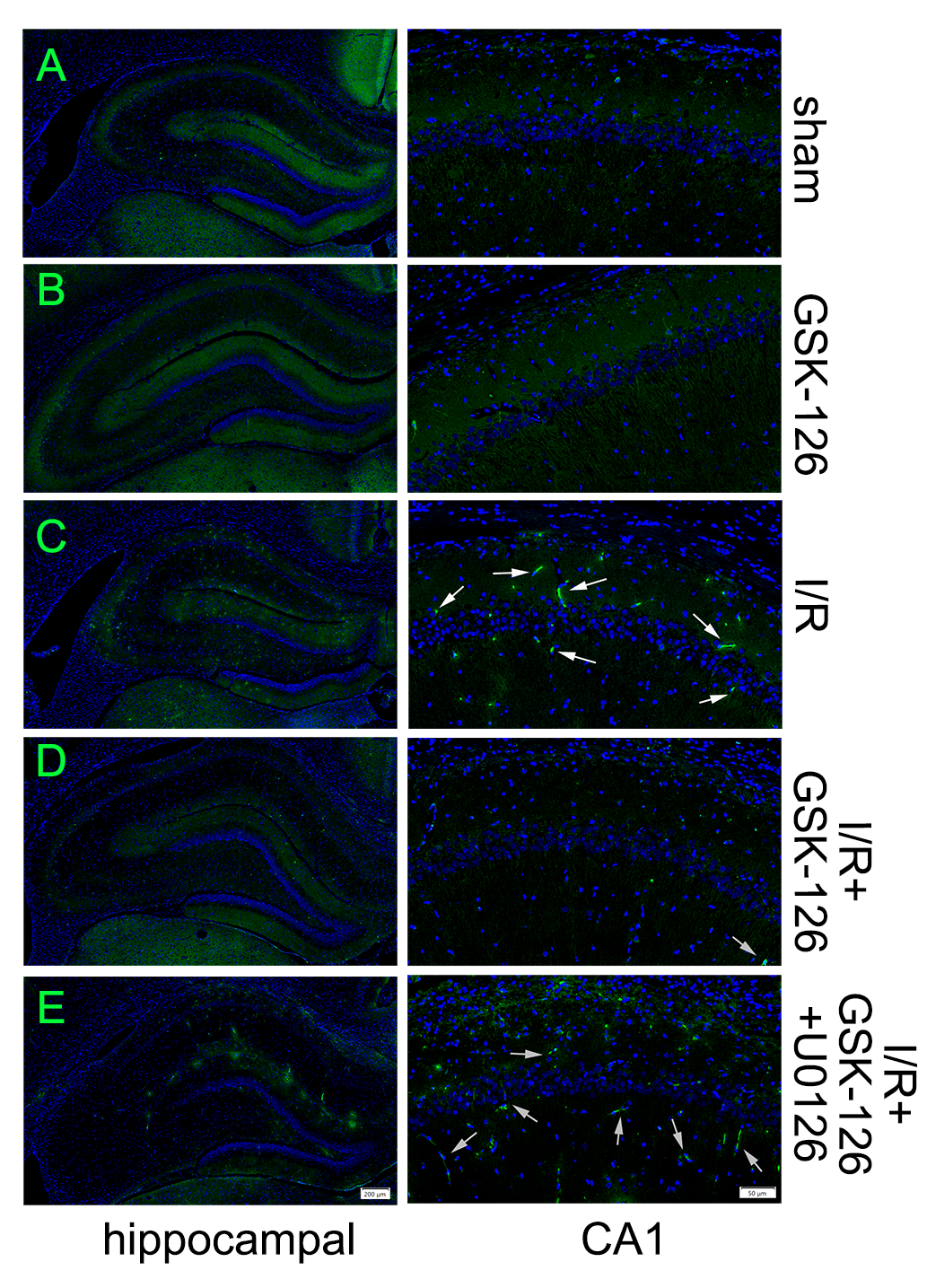

Supplement: Supplementary file 1 — Tunel assay. A. Tunel assay was performed on rat hippocampal section. The left panels show the whole hippocampal section of each group, the scale bar is 200μm.The right panels show the CA1 section of each group, the scale bar is 50 μm. Apoptotic cells were labeled with fuorescein isothiocyanate (green), and all nuclei were stained with DAPI (blue). The arrow points to the apoptotic cells. A. Representive section of sham group. B. Representive section of GSK-126 group. C. Representive section of I/R group. D. Representive section of I/R+ GSK-126 group. E. Representive section of I/R+ GSK-126+U0126 group. N=4. (PNG 3650 kb) [file 12035_2021_2677_Fig7_ESM.png]

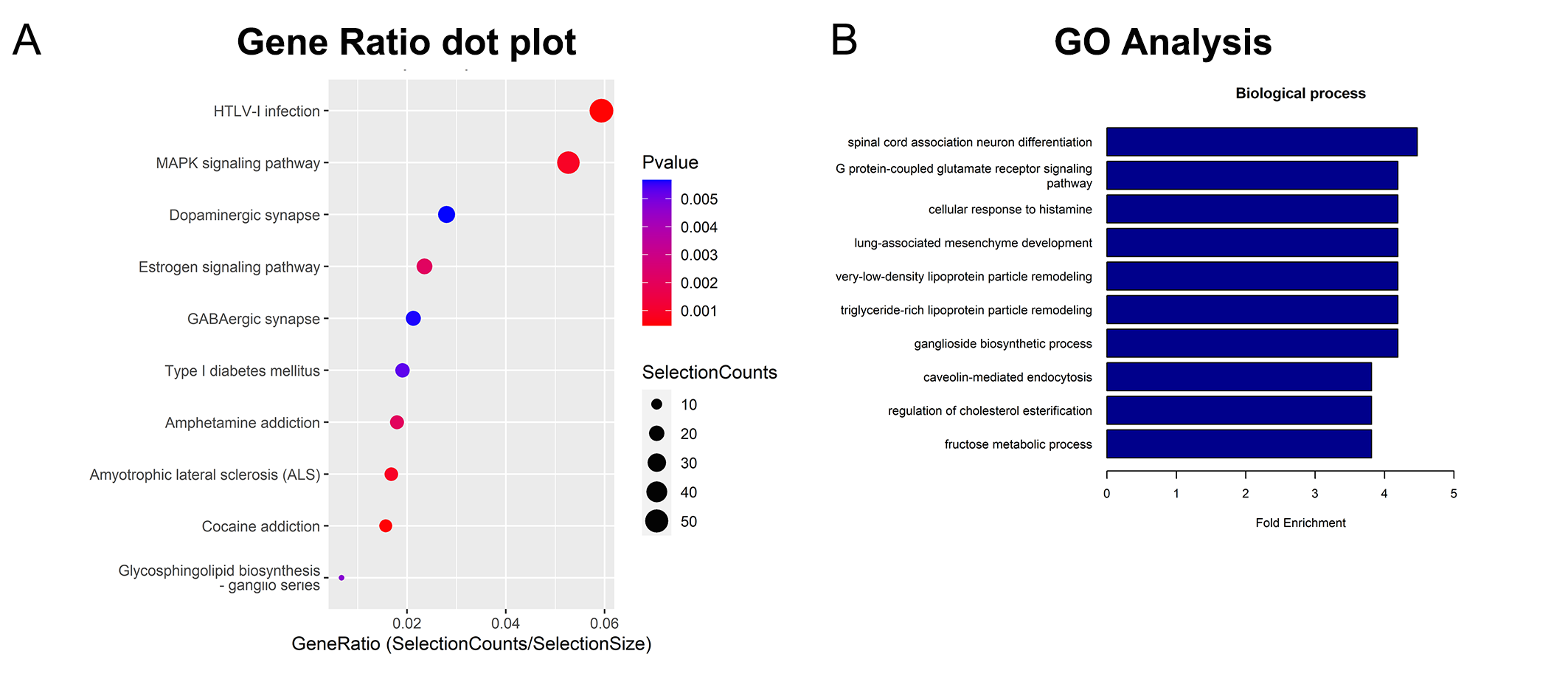

Supplement: Supplementary file 3 — Pathway analysis and GO analysis. A. The gene ratio dot plot of the top 10 significant pathways. B. The top 10 significant GO terms for genes with downregulated enrichment levels of H3K27me3 between I/R rats pretreated with GSK-126 and sham rats. (PNG 226 kb) [file 12035_2021_2677_Fig8_ESM.png]

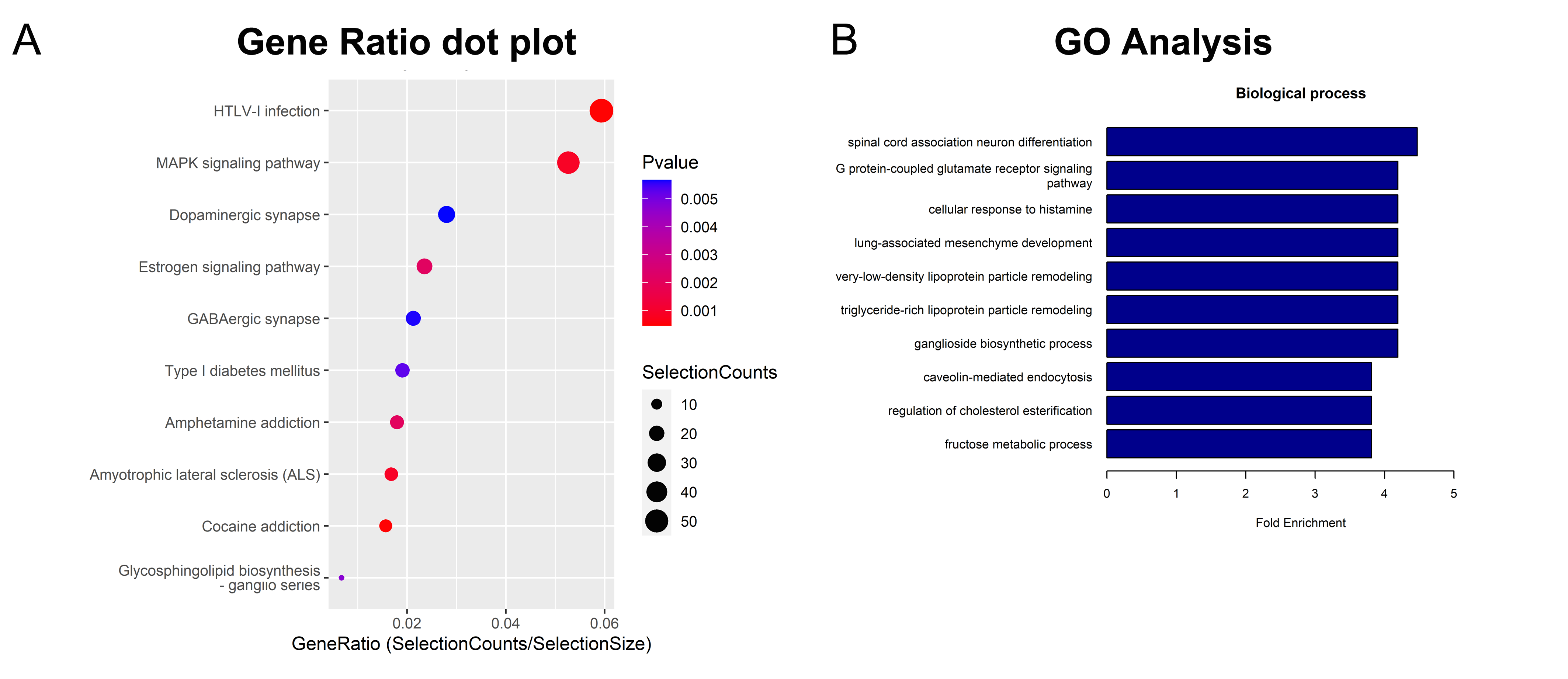

Supplement: Supplementary file 4 — High Resolution Image (TIF 2932 kb) [file 12035_2021_2677_MOESM2_ESM.tif]

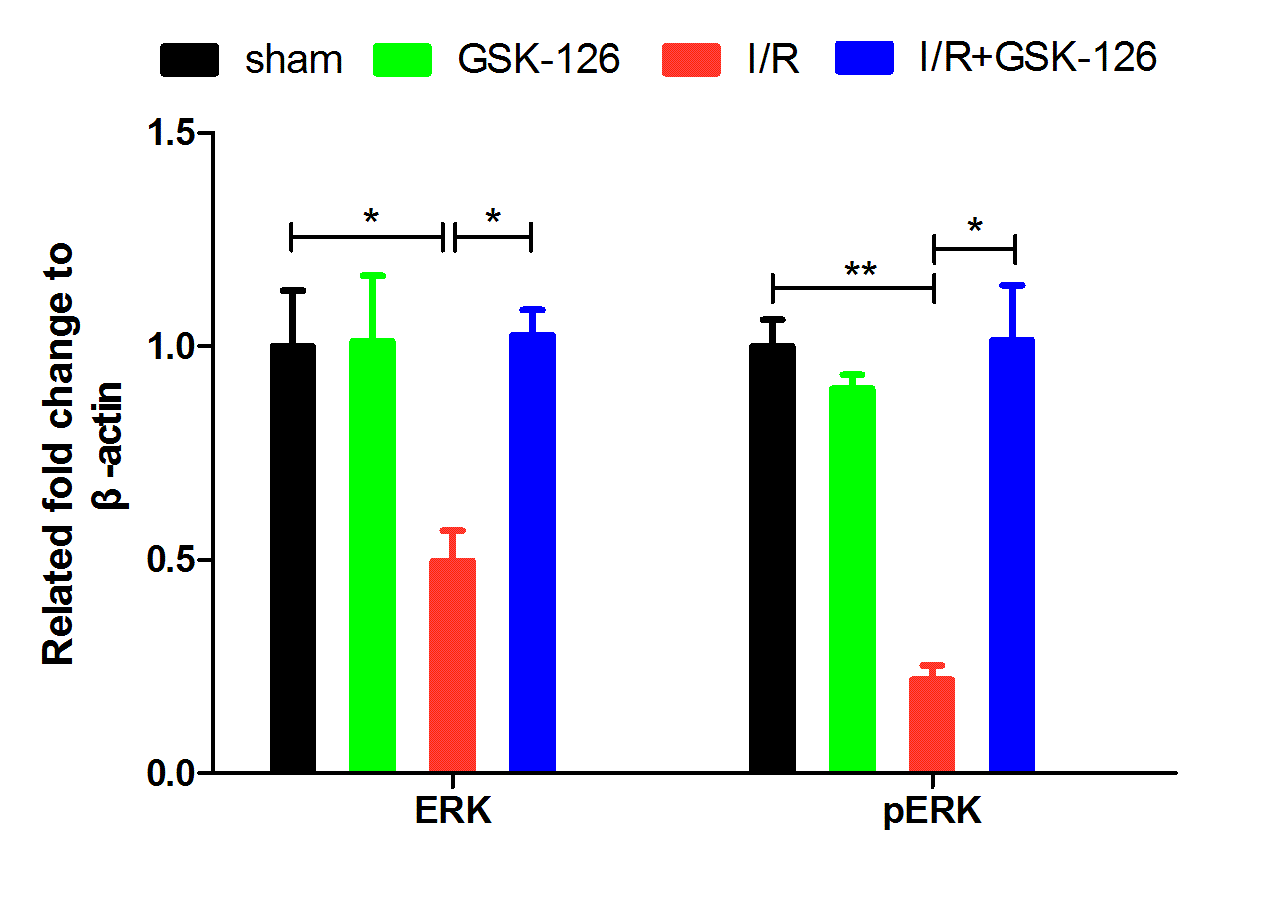

Supplement: Supplementary file 5 — ERK expreesion level in each group. Relative intensity of ERK and pERK illustrated in Figure 4E. *p< 0.05, **p < 0.01. N=3. (PNG 18 kb) [file 12035_2021_2677_Fig9_ESM.png]

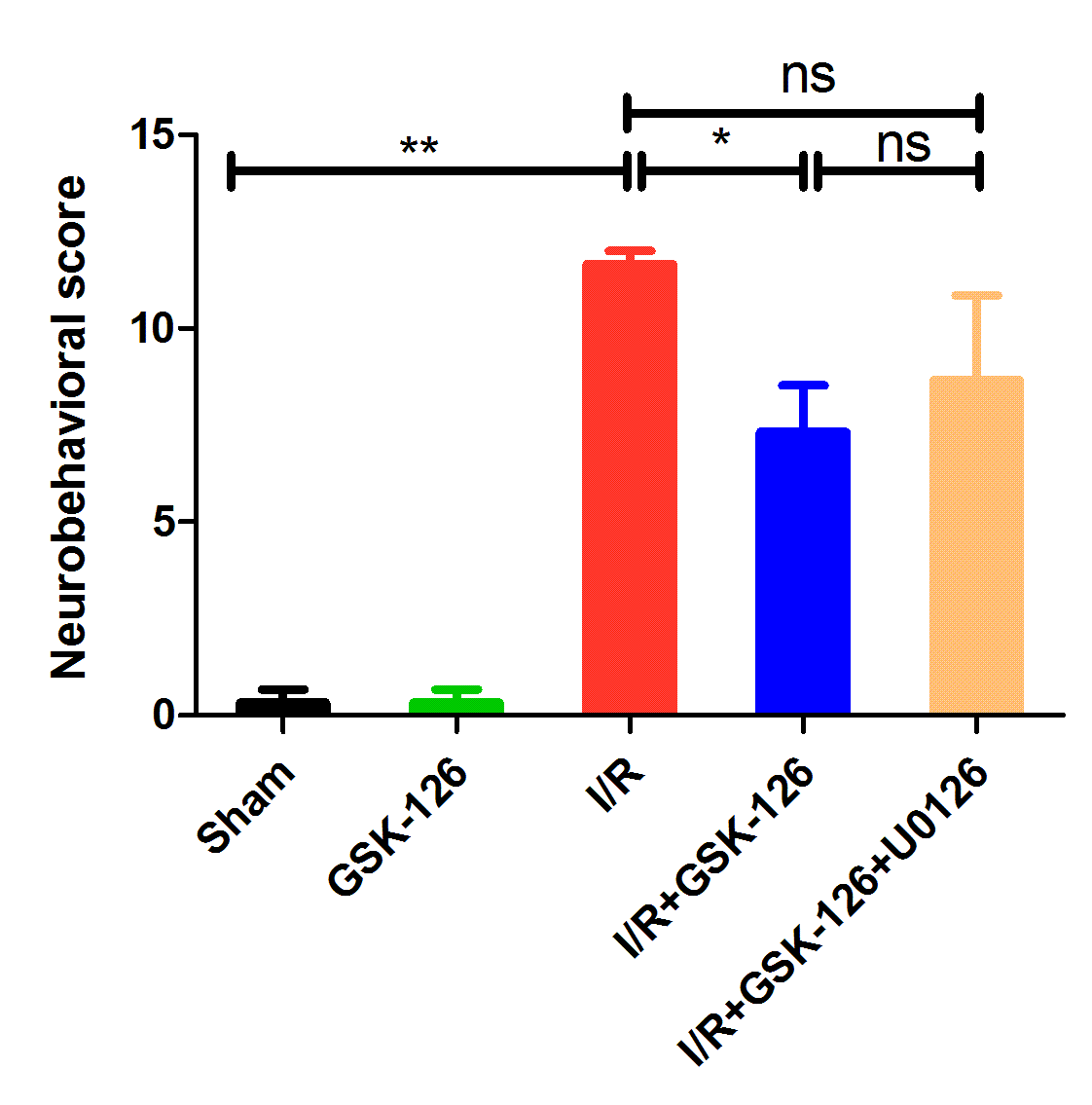

Supplement: Supplementary file 7 — Behavioral evaluation by the mNSS test. ns indicates no significant differences. *p< 0.05, **p < 0.01. N=3. (PNG 24 kb) [file 12035_2021_2677_Fig10_ESM.png]
